# Genipin‐crosslinked decellularized annulus fibrosus hydrogels induces tissue‐specific differentiation of bone mesenchymal stem cells and intervertebral disc regeneration

**DOI:** 10.1002/term.3014

**Published:** 2020-02-12

**Authors:** Yizhong Peng, Donghua Huang, Jinye Li, Sheng Liu, Xiangcheng Qing, Zengwu Shao

**Affiliations:** ^1^ Department of Orthopaedics, Union Hospital, Tongji Medical College Huazhong University of Science and Technology Wuhan China; ^2^ Musculoskeletal Tumor Center, Department of Orthopedics The Second Affiliated Hospital of Zhejiang University School of Medicine Hangzhou China

**Keywords:** annulus fibrosus, decellularized hydrogel, genipin, injectable, in vivo, stem cells

## Abstract

Biomaterial‐based therapy that can restore annulus fibrosus (AF) function in early stage and promote endogenous repair of AF tissues is a promising approach for AF tissue repair. In this study, we established a genipin‐crosslinked decellularized AF hydrogels (g‐DAF‐G) that are injectable and could manifest better in situ formability than noncrosslinked decellularized AF hydrogel, while preserving the capacity of directing differentiation of human bone mesenchymal stem cells (hBMSCs) towards AF cells. Hematoxylin and eosin staining, 4',6‐diamidino‐2‐phenylindole staining, and so forth showed that the majority of cellular components were removed, whereas extracellular matrix and microstructure were largely preserved. The storage modulus increased from 465.5 ± 9.4 Pa to 3.29 ± 0.24 MPa after 0.02% genipin crosslinking of decellularized AF hydrogels (DAF‐G) to form g‐DAF‐G. AF‐specific genes (COL1A1, COL5A1, TNMD, IBSP, FBLN1) were significantly higher in DAF‐G and g‐DAF‐G groups than that in control group after 21 days of culturing. g‐DAF‐G significantly restored nucleus pulposus water content and preserved intervertebral structure in vivo. Summarily, we produced a novel AF regeneration biomaterial, g‐DAF‐G, which exhibited well biocompatibility, great bioactivity, and much higher mechanical strength than DAF‐G. This study will provide an easy and fast therapeutic alternative to repair AF injuries or tears.

## 1 INTRODUCTION

Intervertebral disc degeneration (IVDD), which is a major cause of low back pain (Luoma et al., 2000), has exerted a large socioeconomic burden worldwide. It could be characterized by progressive degenerative damage of intervertebral discs (IVDs) in company with metabolic alterations of other vertebral parts (Ruiz‐Fernandez et al., 2019). Unfortunately, the complicated aetiology/pathogenesis of IVDD largely limits the understanding of this disabling disease and restricts the progression of effective therapies. Annulus fibrosus (AF) defect is a well‐known cause for the initiation and progression of IVDD (Buser, Chung, Abedi, & Wang, 2019). Consistent AF defect after discectomy would lead to disc re‐herniation (Battie et al., 2014) and persistent low back pain. Operative treatment is the only approach that is clinically applied and mostly preferred for AF defect and disc herniation. However, surgical approaches, especially open discectomy, often require vertebral fusion to avoid spinal instability, which decreases spine mobility in a great extent. Also, postoperative complication, including infection, bleeding, unintentional dural puncture/laceration, and so forth, intends to lengthen the hospital duration and even lead to reoperation (Fjeld et al., 2019). For these reasons, an increasing interest has been attracted in biological strategies for IVD regeneration. Among those, biomaterial‐based therapy is a promising approach that can achieve early intervention or postoperative AF repair after AF injuries and prevent further operation.

As it has long been identified that cellular physiology of disc cells is largely affected by the mechanical environment in AF tissues (Vergroesen et al., 2015), many engineered scaffolds have been fabricated for AF biomechanical restoration and pain relief, including natural scaffolds (Zitnay et al., 2018), synthetic polymers (Christiani, Baroncini, Stanzione, & Vernengo, 2019), and their combination (Yang et al., 2017). Among various types of scaffolds, in situ tissue‐derived biomaterials, which closely mimic the prominent features of the native tissue microarchitecture and support an appropriate, tissue‐specific cell phenotype, have their innate advantages in native tissue repair (Bejleri & Davis, 2019). Decellularized matrices are biomaterials produced by removing cellular components of the tissues while retaining most of extracellular matrix (ECM). The process of decellularization not only maintains the original structure and biological function of the native tissues that are essential for cell adhesion, proliferation, and differentiation, but also significantly reduces autoimmunity (Bejleri & Davis, 2019; Xu et al., 2014). Thus, the native tissue‐derived decellularized matrices could provide the innate tissue architecture as well as vital biochemical signaling molecules for the resident cells in various types of tissue regeneration. Unfortunately, despite the several advantages, the IVD‐derived decellularized matrix is limited in direct application of IVD repair due to its nonplasticity.

ECM hydrogels from decellularized IVD tissues are considered to be reliable materials for IVD repair as the injectable property that could fill irregularly shaped defects as well as the matrix structure and biological signals retained from the native source tissue (Mercuri, Gill, & Simionescu, 2011). However, the capacity of decellular IVD‐derived hydrogel in directing cell behavior and promoting in situ tissue repair is poorly understood, especially for decellularized AF (DAF)‐derived hydrogel. Thus, we produced a novel decellularized AF‐derived hydrogel and explored its biocompatibility, cellular fate decision, and ability in IVD defects restoration in vitro/in vivo.

Due to the relatively low mechanical strength of the AF‐derived hydrogel compared with the native AF tissues, genipin, which was extracted from the natural compound geniposide with low toxicity and high biocompatibility, was chosen as a crosslinking agent for the AF‐derived hydrogel. The genipin‐crosslinked DAF hydrogels (g‐DAF‐G) could mimic the mechanical property of native AF tissues better than DAF‐only hydrogel (DAF‐G). Moreover, it has been reported that micromechanical properties determined by the microstructure of materials were effective on cell differentiation induction (Wen et al., 2014). Larger Young modulus (13.4 MPa) was reported to better induce collagen I (Col I) expression than the smaller one (2.5 MPa) in AF‐derived stem/progenitor cells (Guo et al., 2015), suggesting that larger Young modulus is potentially more likely to induce AF‐specific phenotype. Thus, the scaffolds with a relative high stiffness might increase the tendency of resident stem cells into AF‐like differentiation and benefit the AF tissues defects better.

In this study, we developed a genipin‐crosslinked DAF‐G that were injectable and could manifest better in situ formability to overcome the limitations of noncrosslinked DAF‐G. The microstructure, mechanical properties, and biocompatibility of the produced hydrogel in the present study were thoroughly assessed. The differentiation of human bone mesenchymal stem cells (hBMSCs) on the hydrogel and in vivo repair of AF defects rat model was also evaluated. The general schematic design of our study was shown in Figure S1. We hypothesized that the produced hydrogen could effectively direct specific differentiation of hBMSCs towards AF cells and exhibit high efficiency of in vivo AF tissue repair. We hope that our research could offer new insights into the regeneration of IVD.

## 2 MATERIALS AND METHODS

### 2.1 Bovine AF specimen

All animal experiments were managed with the protocol approved by the Animal Experimentation Committee of Huazhong University of Science and Technology. Twenty disc specimens were harvested from four bovine tails that were obtained from a local abattoir. In details, five disc specimens were harvested from each tail by cutting transversely, while surrounding soft tissues and vertebral body were cautiously removed. Then, the outer AF tissues were obtained from each disc and used in the following experiment. Throughout the whole preparation process, the extracted AF samples were regularly sprayed with distilled water to keep them moist.

### 2.2 Preparation of genipin‐crosslinked DAF matrix hydrogels

The dissected AF tissues were cut into 5‐cm pieces for a chemical decellularization process. Briefly, cellular components in AF were sequentially extracted by Triton X‐100 (2%) and sterile water washes at 4°C. After the freeze‐drying processes for five cycles, the AF tissues with Triton X‐100 (2%) were gently shaken on open air shaker for 72 hr at 18°C. Then, the supernatant liquid was removed after 1,200 rpm for 5 min and 1% sodium dodecyl sulfate was applied to wash the AF tissues on a shaker for 72 hr at 18°C. Next, the AF tissues were washed by distilled water for three times (30 min for each time) in order to fully remove the residual sodium dodecyl sulfate solution. For sterilization, the resulting DAF matrix was lyophilized, smashed into powder by a micromill (Thomas Wiley® Mini‐Mill, USA), fumigated in a sealed box by 95% alcohol for 2 hr at 37°C, and irradiated under ultraviolet overnight with ventilation, generating DAF powder (DAF‐P). The obtained DAF‐P (1.5% w/v) was weighed and solubilized in 0.01 mol/L HCl containing pepsin (concentration of pepsin is 1.5 mg/ml) with moderate agitation for 48 hr. The pH of the digested solution (DAF in pepsin–HCl solution) was adjusted to 7.4 using 0.1 mol/L HCL and 0.1 mol/L NaOH, and the salt concentration was adjusted with 10 times phosphate‐buffered saline (PBS; 1/10 of final neutralized volume) to form DAF‐Gs. Genipin (Aladdin, China) dissolved in 75% EtOH was applied to crosslink DAF‐G, with various genipin concentrations (0.01%, 0.02%, and 0.04% w/v), forming g‐DAF‐Gs. Commercialized hydrogel products, Col I (Corning, USA), was utilized in further studies. The final concentration of Col I, DAF, and g‐DAF‐P in the hydrogel was 1.5% w/v. The solution‐to‐gelatin transition was induced by adjusting the temperature to the physiological range (37°C, about 30 min). All procedures were carried out under sterile conditions.

### 2.3 Characterization of fresh AF and DAF‐P

To identify the efficiency of AF decellularization, residual DNA was detected, and histological structures were evaluated by hematoxylin and eosin (HE) staining. To analyze the abundance of ECM components, the concentrations of glycosaminoglycans (GAGs) in fresh AF (FAF) and DAF‐P were detected by tissue GAGs alcian blue colorimetry assay kit (GenMed Scientifics, GMS 19236.2, USA), after lyophilization of FAF and DAF‐P. A standard curve was performed to estimate the concentration of GAGs in each sample. The DNA of FAF and DAF‐P was extracted using the Animal Tissues/Cells Genomic DNA Extraction Kit (Solarbio, Cat#D1700, Beijing, China) and analyzed by NanoDrop microvolume sample retention system (Thermo Scientific). For histological evaluation, FAF and DAF‐P were fixed in 4% formalin, washed three times with distilled water, embedded in paraffin, and sectioned using an ultrathin slicer (Leica EM UC7, Germany). The sections were stained with HE, 4',6‐diamidino‐2‐phenylindole, picrosirius red, improved special Masson trichrome and immunohistochemical staining of collagen 1A1 (COL1A1, A1352, Abclonal) and collagen 2A1 (COL2A1, 15943‐1‐AP, Proteintech), and photographed under a microscope (Olympus, Japan).

### 2.4 Rheological property of hydrogels

To monitor the process of gelation at 37°C (physiological temperature), the pH and ion‐balanced solution was placed on a 20‐mm parallel plate rheometer (Thermo Scientifc, HAAKE MARS III) at 4°C to ensure homogeneous distribution and liquidity of the prehydrogel solutions between the plates. Parameters in the test were set to 1% strain and 1 Hz. To observe the gelation process at 37°C, a dynamic time sweep (10 min) was performed with a fast heating from 4°C to 37°C (200°C/min) at the beginning stage. Storage modulus of each sample was recorded to assess the mechanical characteristics of hydrogels.

### 2.5 SEM observation

FAF, DAF‐P, lyophilized DAF‐G and g‐DAF‐G were fixed in 2.5% glutaraldehyde for 8 h, rinsed in deionized water for 30 min, and then gradiently dehydrated in a series of alcohol solutions (30%, 50%, 75%, 100% ethanol) for half an hour of each rinse. Finally samples were lyophilized and torn to generate the fracture surface. After coating with platinum, the surface of each sample was photographed by scanning electron microscope (SEM; HITACHI S‐4800) at 10 kV. The structural parameters of samples were measured by image analysis software (Image J; National Institutes of Health). More specifically, three SEM pictures from three independent samples for each group were collected for quantitative analysis. Three squares (1/10 width and length of the whole picture) were randomly placed on the picture and the area within the squares were identified as region of interest (ROI). Then, diameters of the fibers included in ROIs were measured, and the largest distance between adjacent fibers included in ROI was measured and defined as fibers distance.

### 2.6 Protein distribution analysis by sodium dodecyl sulfate–polyacrylamide gel

To compare the difference of retained ECM‐related components among DAF‐P, DAF‐G, and g‐DAF‐G, they were lyophilized and ground into powder, weighed, and the protein components were extracted using radioimmunoprecipitation assay lysis buffer (Nanjing Jiancheng Bioengineering institute) containing a protease inhibitor cocktail (Nanjing Jiancheng Bioengineering institute) and a protein phosphatase inhibitor mixture (Nanjing Jiancheng Bioengineering institute). Next, the samples were centrifuged for 25 min at 13,000 rpm and 4°C, and total protein concentration was measured by a BCA Protein Assay Kit (Beyotime Biotechnology, China). Equal amounts of protein in each sample (30 μg) were electrophoretically separated by a 12% sodium dodecyl sulfate–polyacrylamide gel, which was stained with Coomassie Brilliant Blue R250 protein fast stain kit (Nanjing Jiancheng Bioengineering institute) for 1 hr according to the instructions. After image, total protein in the gel was quantified using Image Lab 4.0 software (Bio‐Rad Laboratories, Hercules, CA, USA).

### 2.7 ATR‐FTIR analysis

A Fourier transform infrared (FTIR) Spectrometer (Spectrum 100, Perkin Elmer, USA) equipped with a germanium single bounce micro attenuated total reflection (ATR) objective was applied to measure the infrared radiation (IR) spectrum of each sample. All IR spectra were collected from 75 scans between 500 and 4,000 cm^−1^ at a wavenumber resolution of 5 cm^−1^. The exhibited IR absorbance spectrum for each sample is the mean of spectra collected randomly from three independent locations.

### 2.8 Human bone marrow samples

All experimental protocols involving human tissue and cells were approved by the Medical Ethics Committee of Tongji Medical College, Huazhong University of Science and Technology, China. Human bone marrow specimens were obtained during the femoral head arthroplasty surgery of six volunteer donors (age from 30 to 50 years).

### 2.9 Cell extraction

hBMSCs were isolated from bone marrow aspirates and cultured in vitro as follows: 10 ml of bone marrow was diluted (1:1) with human MSC complete medium (Cyagen, USA) and loaded over Percoll (Sigma, USA) for density gradient centrifugation. Mononucleated cells were obtained from the interface after centrifugation (900g, 25 min) and washed by PBS (Bosterbio, USA) twice. Then, cells were resuspended in human MSC complete medium and seeded in T25 flasks (Corning, USA) at a density of 1.0 × 10^5^/cm^2^ at 37°C and 5% CO_2_. After 48 hr, medium change was conducted to remove nonadherent cells. The adherent cells were cultured for about 2 weeks until cell clones reached about 75–85% confluence, then digested with 0.25% trypsin‐0.02% ethylenediaminetetraacetic acid (Sigma, USA) and finally subcultured at a density of 1.0 × 10^5^ cells/cm^2^ in new T25 flasks. The third generation of hBMSCs was used throughout the whole experiments. The neutralized hydrogels were uniformly spread in a six‐well culture plate for 100 μl per well and placed in cell incubator (37°C) for 30 min for gelation. A thin hydrogel layer coating was created after gelation, and the suspension cells in the third generation were counted and plated onto the surface of hydrogel scaffolds with Dulbecco's modified Eagle medium/ha F‐12 (Gibco, USA) containing 1% fetal bovine serum (Gibco, USA) and 1% penicillin/streptomycin (Beyotime, China) at 37°C and 5% CO_2_. The cells cultured on coating hydrogels were used for cell viability assessment and AF specific gene detection.

### 2.10 Assessment of cell viability

Cell compatibility assays were conducted using Cell Counting Kit‐8 (CCK‐8, KeyGEN BioTECH, China). Cells were plated on 96‐well plates (control) or the Col I, DAF‐G, or g‐DAF‐G precoated 96‐well plates (2,000 cells per well). After 1, 7, 14, and 21 days, 100‐μl CCK‐8 working solution (CCK‐8 reagent: serum‐free medium = 1:10) was added into each well of the plate and incubated for 2 hr. Then, to avoid the influence of the bottom hydrogel scaffolds on the absorbency, the CCK‐8 working solution of each well was successively transferred to a new blank 96‐well plate. Finally, absorbency, indicating cell viability, was detected at 450 nm using a spectrophotometer (ELx808 Absorbance Microplate Reader, Bio‐Tek, USA). In addition, Live‐Dead cell staining kit (KeyGEN BioTECH, China) was carried out by treating cells with calcein AM and PI for 10 min, after hBMSCs were cultured on 96‐well plates (control) or the Col I, DAF‐G, or g‐DAF‐G precoated 96‐well plates for 14 or 21 days, and then observing the image by a fluorescence microscope (Olympus, IX73, Japan).

### 2.11 LDH assay

The 96‐well plate was precoated with none (control), 20‐μl DAF‐G and 20‐μl g‐DAF‐G of various genipin concentrations (0.01%, 0.02%, and 0.04%). hBMSCs were seeded at a density of 2,000 cells per well, and lactate dehydrogenase (LDH) Cytotoxicity Assay Kit (Beyotime, Shanghai, China) was utilized to evaluate the cytotoxicity of the various genipin concentration on hBMSCs at Day 21 according to the protocol. LDH release activity was calculated from optical density value measured by spectrophotometer (ELx808 Absorbance Microplate Reader, Bio‐Tek, USA).

### 2.12 Quantitative real‐time RT‐PCR analysis

Gene expression was measured by reverse transcription polymerase chain reaction (RT‐PCR) after 7 or 21 days of hydrogel culturing. Total RNA from hBMSCs was harvested using the TRIZOL reagent (Invitrogen, USA). Complementary DNA was synthesized using the RevertAid™ First Strand cDNA Synthesis Kit (K1622; Fermentas) and oligo (dT) primers (15 min at 37°C and 5 s at 85°C) on a RT‐PCR system (Eastwin Life Science, Beijing, China). RT‐PCR was carried out with a Bio‐Rad CFX96™ Real‐Time System using the SsoFast™ EvaGreen Supermix Kit (Bio‐Rad). Primer sequences for collagen‐1A1, collagen‐5A1, integrin‐binding sialoprotein (IBSP), fibulin‐1 (FBLN1), tenomodulin (TNMD), and glyceraldehyde‐3‐phosphate dehydrogenase (the control) were listed in Table 1. The relative expression levels were analyzed by the 2^−ΔΔCt^ method and normalized to the control.

**Table 1:** Summary of primers used in quantitative reverse transcription polymerase chain reaction

Gene	Sequence (5'‐3')	
COL1A1	Forward primer	GATTGACCCCAACCAAGGC
	Reverse primer	GAATCCATCGGTCATGCTCT
COL5A1	Forward primer	CCGATTGGCTACCCAGGTC
	Reverse primer	CACCCTTGATGCCCATGTCT
TNMD	Forward primer	TTGGTATCCTGGCCCTAA
	Reverse primer	CAGTGCCATTTCCGCTTC
IBSP	Forward primer	AGGGCAGTAGTGACTCATCCG
	Reverse primer	AGCCCAGTGTTGTAGCAGAAAG
FBLN1	Forward primer	TCTCTGTGGATGGCAGGTCA
	Reverse primer	ACACTGGTAGGAGCCGTAGA
GAPDH	Forward primer	AATCCCATCACCATCTTCCAG
	Reverse primer	GAGCCCCAGCCTTCTCCAT

Abbreviations: FBLN1, fibulin‐1; GAPDH, glyceraldehyde 3‐phosphate dehydrogenase; IBSP, integrin‐binding sialoprotein; TNMD, tenomodulin.

### 2.13 Rat tail acupuncture degenerative model

Sixteen male Sprague Dawley rats around 300 g (8–12 weeks old) were used for the in vivo experiments. The surgical procedures were described as follows: After anaesthetized by 1% pentobarbital sodium (ml/g) via intraperitoneal injection. The coccygeal vertebrae Co4/Co5 and Co5/Co6 were located by manual palpation and counting and confirmed by a trial radiograph. The IVDs were punctured by a 18‐G sterile needle with an outer diameter of 1.27 mm at a depth from the skin to near the border of the inner AF and nucleus pulposus (NP) tissues with NP intact. Then, the needle was rotated 360° and held for 30 s. Next, 10‐μl physiological saline or the neutralized prehydrogel solution was injected into the predisposed defect of AF tissues by microsyringes (Hamilton injection needle) attached to 20‐G needles. The concentration of genipin in g‐DAF‐G was 0.02% (w/v). The injection of hydrogels was delayed for approximately 20 min, to allow for pregelation (forming Col I, DAF‐G, and g‐DAF‐G) and avoid diffusion or leakage. The rats were randomly divided into four groups for different treatment (four rats in each group): the degeneration group (with needle puncture and physiological saline injection); the Col I group (with needle puncture and Col I injection); the DAF‐G group (with needle puncture and DAF‐G injection); and the g‐DAF‐G group (with needle puncture and g‐DAF‐G injection). The process of gelation could be observed in situ after injection for 30 min. A semitransparent gel was formed in situ for the Col I and DAF‐G groups, whereas a blue gel was coagulated for the g‐DAF‐G group. Magnetic resonance imaging (MRI), and histological analysis were performed to assess the repair effect of hydrogel on AF defects in each group.

### 2.14 MRI procedures and data processing

At 4 and 8 weeks after injection, all rats were anesthetized with 1% pentobarbital sodium (ml/g) and were put on examination plate with their tails straightened. T2‐weighted sections in the sagittal plane were imaged by MRI (MAGNETOM Avanto, 1.5 T, Germany) to analyze the water content and the structure of the target disc. ImageJ was used for quantitative analysis of the image slices to measure the relative NP water content by the ratio of punctured disc to adjacent intact disc. The alterations of the NP tissues were also assessed by modified Pfirrmann disc degeneration grading system (Che et al., 2019). Two researchers carried out the assessment independently.

### 2.15 Histological analysis

After injection for 8 weeks, target disc specimens in each group were harvested by euthanizing the rats with 1% pentobarbital sodium (ml/g). The disc samples were rinsed by PBS buffer solution, fixed in 4% paraformaldehyde, embedded in paraffin, and cut into 5.0 μm per section (Thermo#shandon finesse 325). Next, disc specimens were stained with HE and safranin O–fast green (S&O). The cellularity and morphology of the IVD tissues were assessed by two authors separately according to the scoring criteria described by Han et al. (2008)

### 2.16 Statistical analysis

The outcomes were presented as the mean ± standard deviation (SD), and a value of p < 0.05 was considered statistically significant. The statistical differences between treatment groups were evaluated by one‐way analysis of variance, following the Tukey post hoc test. All experiments were repeated at least three times using independent samples. Statistical analyses were performed using the SPSS software package (IBM SPSS software package 18.0). All the statistical chart was drawn by GraphPad Prism 6 software (GraphPad Software Inc., San Diego, CA).

## 3 RESULTS

### 3.1 FAF and DAF‐P characterization

**Figure 1:**
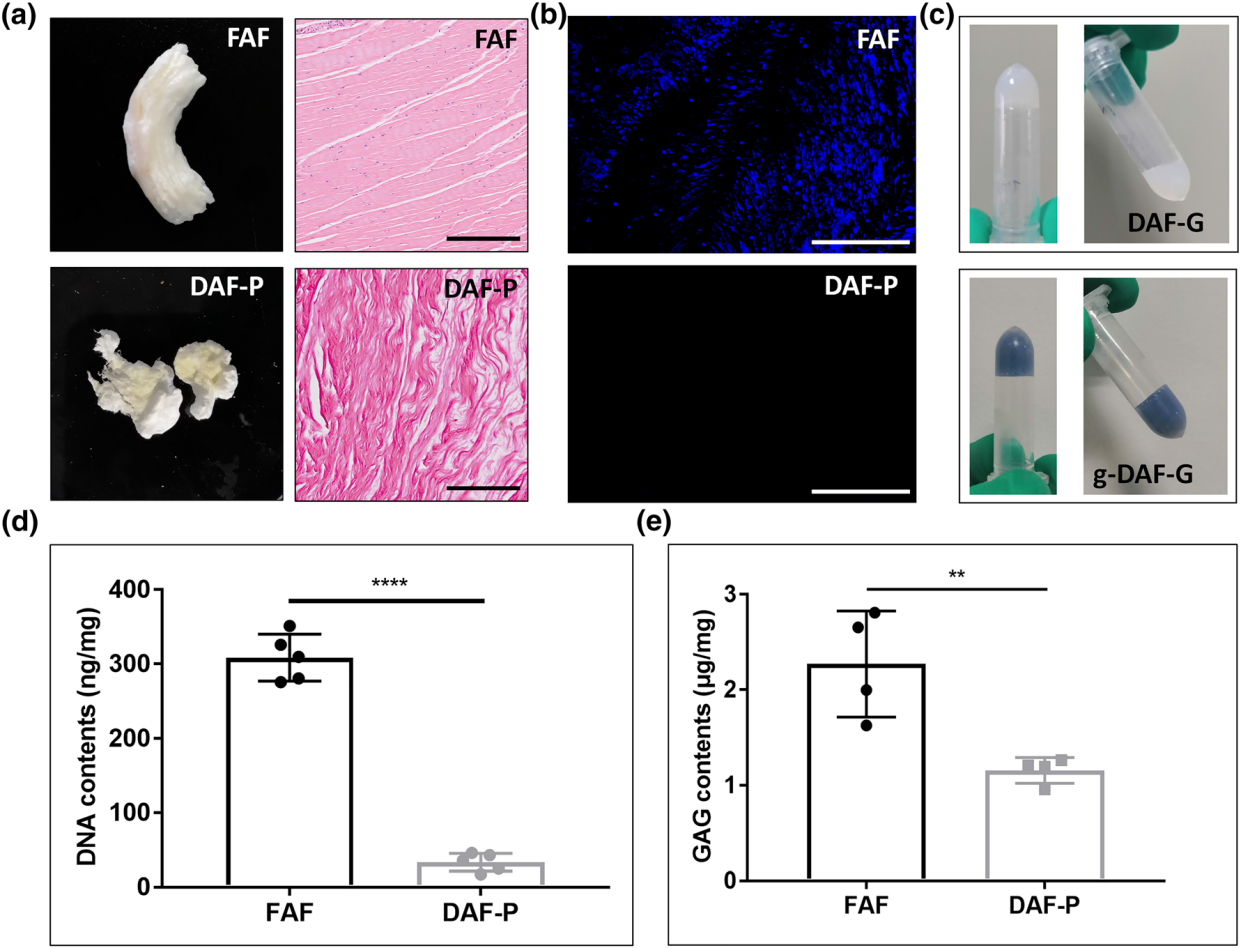
Fabrication of decellularized annulus fibrosus (AF) hydrogel and evaluation of decellularization efficiency. (a) General appearance and hematoxylin and eosin staining of fresh AF (FAF) and decellularized AF powder (DAF‐P). (b) 4',6‐Diamidino‐2‐phenylindole staining revealed significant removal of cellular components after decellularization. (c) Decellularized AF hydrogels (DAF‐G) appealed to be semitransparent and oyster milk, and genipin‐crosslinked decellularized AF hydrogels (g‐DAF‐G) turned blue after gelation and was more stable. Black bar = 200 μm, white bar = 500 μm. Quantitative analysis of (d) DNA and (e) glycosaminoglycans suggested high efficiency of decellularization and maintenance of extracellular component. Data are presented as the *M* ± *SD* of four or five independent experiments (**p < .01 and ****p < .0001 between two groups) [Colour figure can be viewed at http://wileyonlinelibrary.com]

After decellularization, the DAF tissue became white and porous, and HE staining showed an absence of nuclei and a loose‐layered structure in histological sections (Figure 1a). Also, 4',6‐diamidino‐2‐phenylindole staining suggested obviously less positive staining after decellularization, indicating efficient removal of DNA. (Figure 1b). During gelation, the genipin‐crosslinked AF hydrogels gradually turned into blue, and AF hydrogels formed a semitransparent gel (37°C, pH 7.3–7.5; Figure 1c). Residual DNA content for FAF and DAF‐P were 360 ± 31 and 40 ± 4 ng/mg, respectively. DNA derived from DAF‐P was lower than the internationally recognized criterion of 50 ng/mg (Crapo et al., 2012; Figure 1d). The outcomes of the GAGs content for FAF and DAF‐P showed 2.27 ± 0.56 and 1.16 ± 0.13 μg/mg, respectively (Figure 1e). Also, as what COL1A1, COL2A1, picrosirius red, and masson staining suggested, the arrangement of ECM collagens became a little bit loose, and the staining intensity, indicating collagen content, did not get altered significantly after decellularization (Figure S2). Therefore, our protocol for removing cellular component while preserving ECM is reliable and efficient.

### 3.2 Ideal concentration of genipin and biocompatibility of g‐DAF‐G

**Figure 2:**
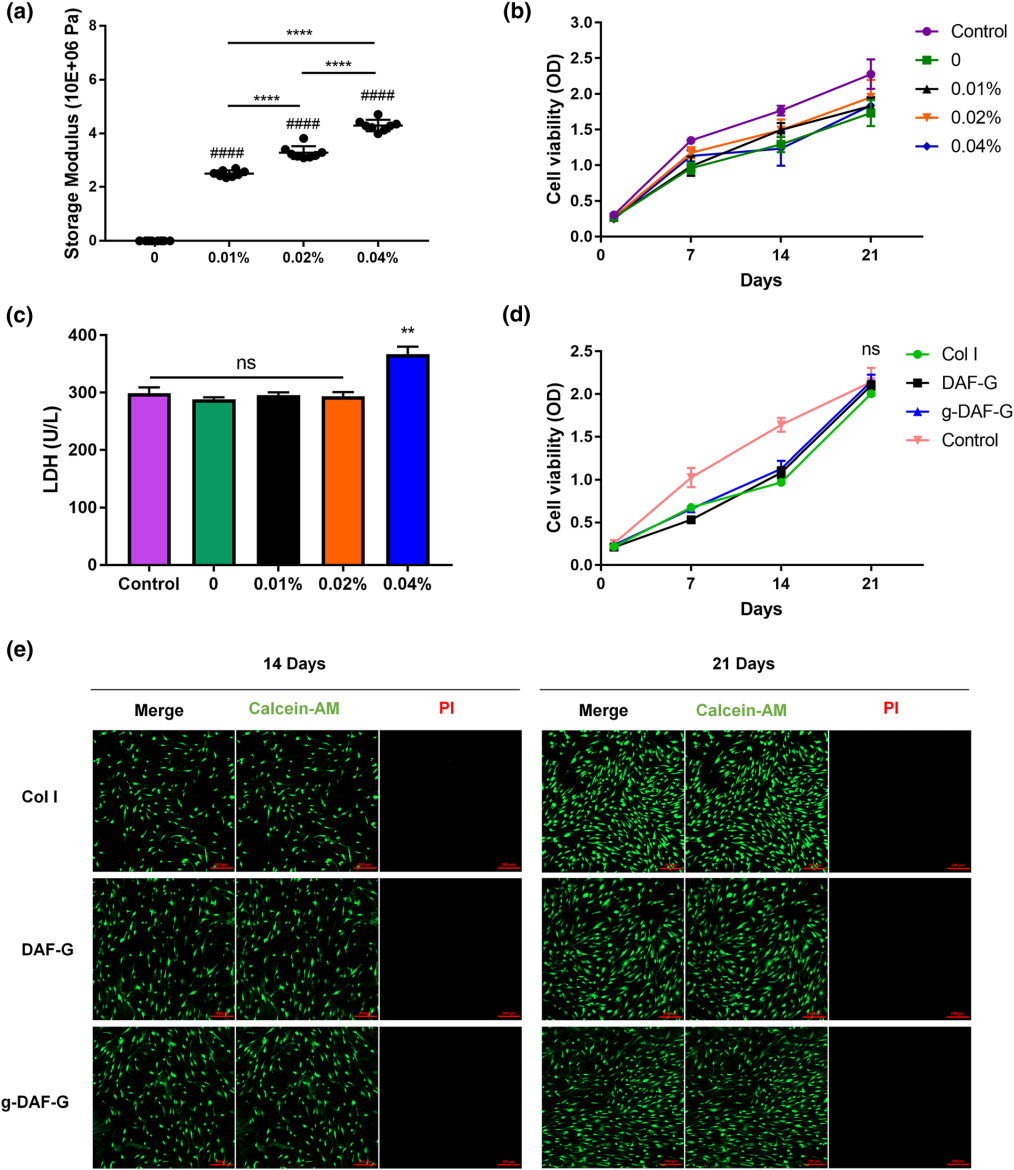
Effects of various concentration of genipin on cytotoxicity, mechanical properties, and proliferation. (a) Genipin crosslinking improved storage modulus of decellularized annulus fibrosus hydrogels. (b) Increased concentration of genipin did not significantly impair cell viability. (c) 0.04% genipin induced more LDH release. (d) Human bone mesenchymal stem cells (hBMSCs) cultured on hydrogels or culture plates showed similar cell viability on Day 21. Data are presented as the *M* ± *SD* of three independent experiments (####*p* < .0001 between genipin‐crosslinked groups and genipin absent group; **p < .01, ****p < .0001 between indicated two groups; ns, no significance). (e) Live and dead staining of hBMSCs cultured on Col I, DAF‐G, and g‐DAF‐G for 14 and 21 days. DAF‐G, decellularized annulus fibrosus hydrogels; g‐DAF‐G, genipin‐crosslinked decellularized annulus fibrosus hydrogels; LDH, lactate dehydrogenase; OD, optical density [Colour figure can be viewed at http://wileyonlinelibrary.com]

The storage modulus (G′) of g‐DAF‐G was significantly larger than DAF‐G and gradually elevated as the concentration of genipin increased. More specifically, the storage modulus of DAF‐G and g‐DAF‐G with 0.01%, 0.02%, and 0.04% genipin were 465.51 ± 9.43 Pa and 2.57 ± 0.12, 3.29 ± 0.24, and 4.34 ± 0.21 MPa (Figure 2a). hBMSCs cultured on DAF‐G and g‐DAF‐G with various genipin concentration exhibited slightly declined cell viability, compared with hBMSCs cultured on 96‐well plates (Figure 2b). However, cells released more LDH when cultured on g‐DAF‐G with 0.04% genipin, suggesting cell toxicity of excessive genipin (Figure 2c). Thus, 0.02% genipin was chosen for further studies. The absorbance declined a bit in Col I, DAF‐G, and g‐DAF‐G groups as compared with the control (hBMSCs seeded directly on culture plate without hydrogel) on Days 7 and 14, whereas no significance was observed between the four groups on Day 21 (Figure 2d). This indicated that the DAF‐G and g‐DAF‐G coating would not generate an unfavorable influence on cell proliferation for a long‐term culture. The temporary decrease of absorbance on Days 7 and 14 might be attributed to the inadaptation of hBMSCs to the materials. In addition, the results of live‐dead cell staining showed the spindle‐shaped cells evenly distributed on the visual field for Col I, DAF‐G, and g‐DAF‐G groups (Figure 2e), which indicated a good biocompatibility of these biomaterials.

### 3.3 Nanofibrous structure of FAF, DAF‐P, DAF‐G, and g‐DAF‐G

**Figure 3:**
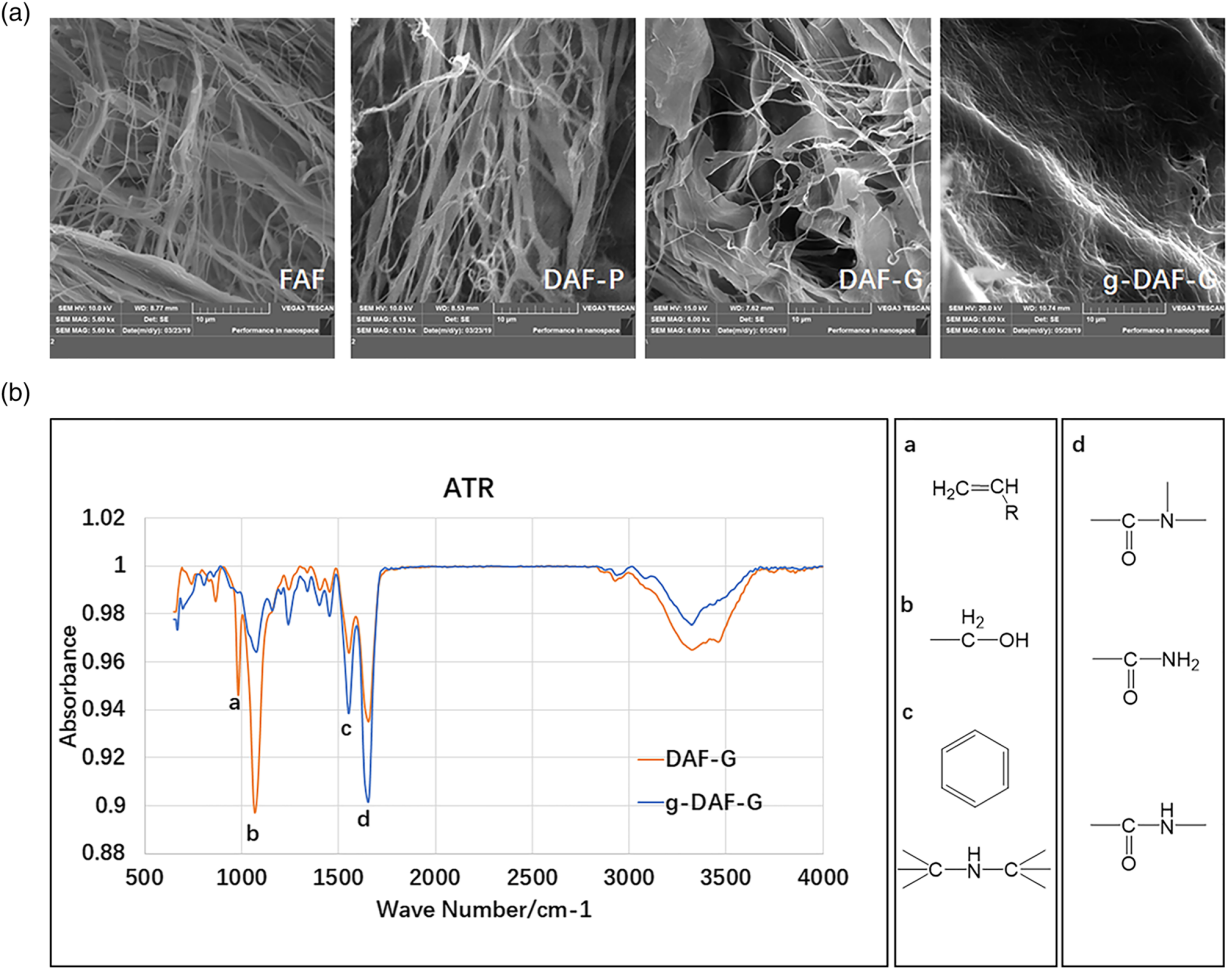
Ultrastructure of materials and chemical bonding formation during gelation. (a) Scanning electron microscope analysis of nanofibrous structure of FAF, DAF‐P, DAF‐G, and g‐DAF‐G. (b) ATR‐Fourier transform infrared analysis detected increased amide bond formation at around 1,600 cm^−1^, and hydroxyl bond at around 1,050 cm^−1^ decreased in g‐DAF‐G compared with DAF‐G. ATR, attenuated total reflection; DAF‐G, decellularized annulus fibrosus hydrogels; FAF, fresh annulus fibrosus; g‐DAF‐G, genipin‐crosslinked decellularized annulus fibrosus hydrogels [Colour figure can be viewed at http://wileyonlinelibrary.com]

SEM analysis revealed that DAF‐P preserved a similar porous structure and assembled fibers as FAF (Figure 3a). After gelation, the DAF‐G existed in microfilm structure with larger distance between fibers. As crosslinked with genipin, the hydrogels (g‐DAF‐G) exhibited a denser fibrous structure and distance between fibers significantly shrunk (Table 2). Moreover, the results of ATR‐FTIR showed that more amide bond (absorption peaks at around 1,600 cm^−1^) formed in g‐DAF‐G compared with DAF‐G, indicating the peptide chain extension after crosslinked with genipin (Figure 3b).

**Table 2:** Quantitative analysis of biomaterials ultrostructure

Biomaterials	Fibro diameters/nm	Distance between fibers/nm
FAF	683.40 ± 228.35	2631.68 ± 801.19
DAF	719.12 ± 204.95	1985.67 ± 1198.91
DAF‐G	418.65 ± 92.3	3794.56 ± 1326.7
g‐DAF‐G	406.74 ± 115.23	1100.13 ± 440.98

Abbreviations: DAF, decellularized annulus fibrosus; DAF‐G, decellularized annulus fibrosus hydrogel; FAF, fresh annulus fibrosus; g‐DAF‐G, genipin‐crosslinked decellularized annulus fibrosus hydrogels.

### 3.4 Difference in the protein composition among DAF‐P, DAF‐G, and g‐DAF‐G

The protein compositions of DAF‐P, DAF‐G, and g‐DAF‐G were analyzed and compared. Coomassie bright blue staining in the study was only applied to roughly analyze the protein component of the proteins in the biomaterials. The outcomes suggested that the proteins in the DAF‐P, DAF‐G, and g‐DAF‐G mainly ranged from 100–130 kDa. We speculated that the mixture of Col I (approximately 130 kDa), Collagen VI (approximately 108 kDa), and other unknown matrix proteins collectively contributed to the 100‐ to 130‐kDa bands. The increased intensity of 100‐ to 130‐kDa bands and decreased intensity of <55 kDa bands in g‐DAF‐G and DAF‐G groups than DAF‐P might result from the process of gelation, which combined some small protein molecules (<55 kDa) into large protein molecules (100–130 kDa) by intermolecular interactions. The existence of genipin further strengthens the gelation process and leads to the highest intensity of 100–130 kDa. More studies in the future need to be conducted to clarify this point.

### 3.5 AF‐specific gene expression

**Figure 4:**
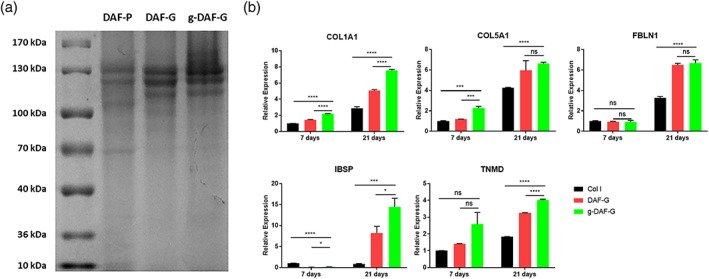
Protein component of decellularized annulus fibrosus materials and their differentiation promoting capacity. (a) Coomassie bright blue staining analysis of DAF‐P, DAF‐G, and g‐DAF‐G. (b) Gene expression levels of human bone mesenchymal stem cells cultured on Col I, DAF‐G, genipin‐cross‐linked DAF‐G. COL1A1, COL5A1, FBLN1, IBSP, and TNMD were measured on Days 14 and 21. Data are presented as the *M* ± *SD* of three independent experiments (*p < .05, ***p < .001, ****p < .0001 between indicated two groups; ns, no significance). DAF‐G, decellularized annulus fibrosus hydrogels; FBLN1, fibulin‐1; g‐DAF‐G, genipin‐crosslinked decellularized annulus fibrosus hydrogels; IBSP, integrin‐binding sialoprotein; TNMD, tenomodulin [Colour figure can be viewed at http://wileyonlinelibrary.com]

For 2D culture, the level of expression of COL1A1, COL5A1, FBLN1, IBSP, and TNMD was significantly higher in the DAF‐G and g‐DAF‐G groups than Col I group on Day 21, which indicated the potential induction of directing specific differentiation of hBMSCs towards AF cells on DAF‐G and g‐DAF‐G hydrogels (Figure 4b). Interestingly, COL1A1, TNMD, and IBSP were observed to be higher in the g‐DAF‐G groups as compared with the DAF‐G group on Day 21(Figure 4b), which implied that the addition of genipin might strengthen AF‐lineage differentiation. RNA quantification indicated that RNA extracted from each group was abundant (Table S1).

### 3.6 In vivo AF repair using Col I, DAF‐G, and g‐DAF‐G

**Figure 5:**
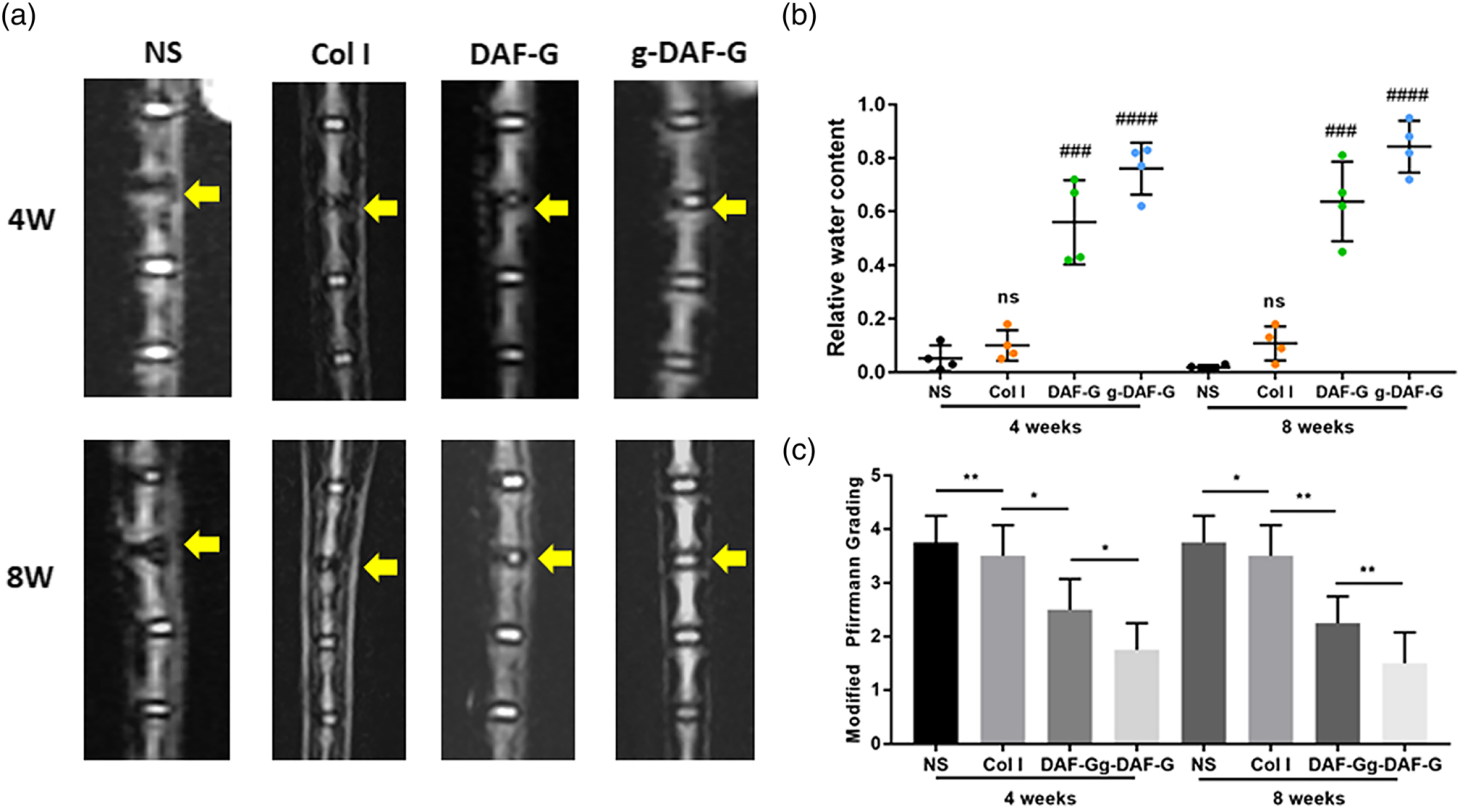
In vivo repairment effects of DAF‐G and g‐DAF‐G on needle puncture annulus fibrosus defect rat model. (a) Representative T2 magnetic resonance imaging scans of the rat Co4‐5 intervertebral disc generations at 4 and 8 weeks after injection. (b) Quantitative analysis of water content within nucleus pulposus tissues. (c) Evaluation of materials in vivo repair efficiency by modified Pfirrmann grading. (###*p* < .001, ####*p* < .0001 between NS group and other groups; *p < .05, **p < .01 between indicated two groups; ns, no significance). DAF‐G, decellularized annulus fibrosus hydrogels; g‐DAF‐G, genipin‐crosslinked decellularized annulus fibrosus hydrogels; NS, normal saline [Colour figure can be viewed at http://wileyonlinelibrary.com]

**Figure 6:**
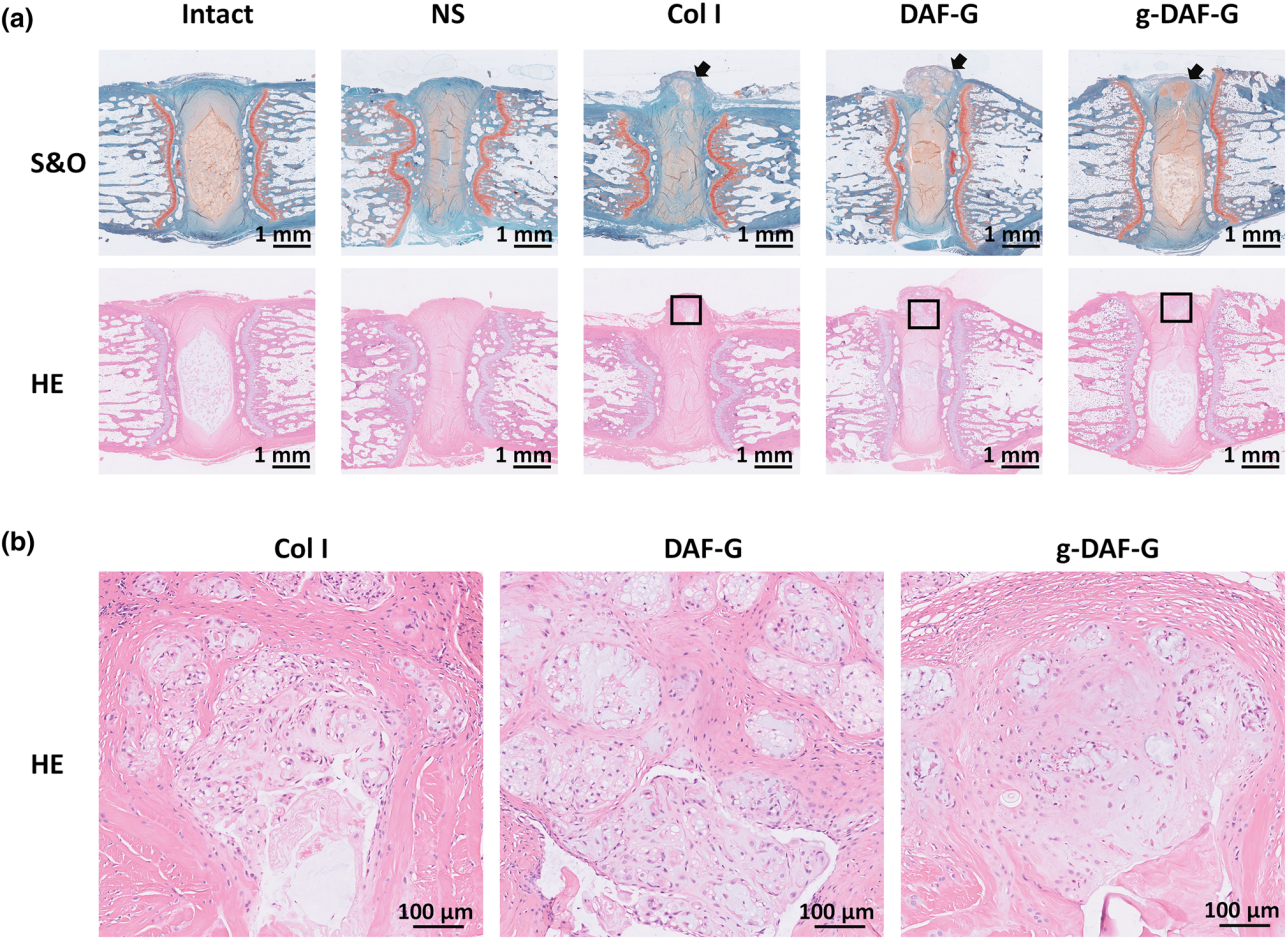
Representative HE and S&O staining of coccygeal disc at 4 and 8 weeks after injection. (a) HE and S&O staining indicated significant structure regeneration in DAF‐G and g‐DAF‐G groups, of which g‐DAF‐G was more efficient. (b) Detailed structure of injected hydrogels showed obvious cellular migration into the gels. Scale bar = 1 mm or 100 μm. Black arrows showed the predisposed defect of annulus fibrosus tissues. Black squares marked the hydrogel region. DAF‐G, decellularized annulus fibrosus hydrogels; g‐DAF‐G, genipin‐crosslinked decellularized annulus fibrosus hydrogels; HE, hematoxylin and eosin; NS, normal saline; S&O, safranin O–fast green [Colour figure can be viewed at http://wileyonlinelibrary.com]

The rat tail acupuncture degenerative model was constructed to assess the regenerative effects of Col I, DAF‐G, or g‐DAF‐G in vivo. The T2‐weighted images showed that intervertebral space became narrower and the signal intensity of the NP region sharply declined in normal saline (NS) group as compared with the adjacent and intact discs (Figure 5a). Quantitative analysis showed that relative water content of DAF‐G and g‐DAF‐G groups were larger than the Col I gel and the NS groups (Figure 5b), indicating a potential regenerative function of AF‐derived hydrogels on AF tissue defect. Moreover, the restorative effect analyzed by quantitative analysis and modified Pfirrmann grading was better in g‐DAF‐G than DAF‐G group (Figure 5b,c), revealing that the mechanical strength improved by genipin might be a vital factor in AF repair. In accordance with expectation, HE and S&O staining showed that declined proteoglycans content and irregular distribution of ECM in AF tissues were observed in the NS, Col I, and DNP‐G groups (Figure 6a). Though AF collagens were disconnected, the g‐DAF‐G hydrogels successfully filled up the gap. The g‐DAF‐G structure was more intense and well aligned, and adjacent AF structure damage in g‐DAF‐G group was significantly slighter than other groups. Similarly, HE and S&O staining also showed much less NP area, and the number of cells was significantly decreased in the NS and Col I groups than g‐DAF‐G and DAF‐G groups. Also, the hydrogels filled with disc cells and abundant ECM were clearly identified on the AF defects (Figure 6b). Altogether, these results demonstrated a satisfied regenerative function of DAF‐G and g‐DAF‐G on AF defect model in vivo.

## 4 DISCUSSION

Increasing interest has aroused in decellularized ECM during the past decades for its fancy biocompatibility and biofunction (Cheng, Solorio, & Alsberg, 2014). This unique biomaterial is made by removing native cells, while largely preserving the ECM components (immunologically conserved among species; Cheng et al., 2014), including collagen, GAGs, proteoglycans, and growth factors (Saldin, Cramer, Velankar, White, & Badylak, 2017). Therefore, decellularized ECM retains native microstructure and biocompatibility, but without inflammatory and immune responses, thus decreasing the risk of implant rejection (Yuan et al., 2013). Recently, injectable materials have received more attention, due to their facility of transplantation, well malleability, and less local damage (Tu et al., 2019). This kind of material can be used for minimally invasive treatment for AF tear before NP herniation, which can significantly reduce the need for surgery or reduce the recurrence after resection of NP in the future (Guterl et al., 2013). AF, composed of bundles of collagen fibers, forms a multiple lamellae microstructure, which endows AF with the capability for resisting shear deformation and maintaining NP integrity (Guerin & Elliott, 2006). Therefore, ideal mechanical strength is required for novel biomaterials for AF repair. However, the process of producing injectable hydrogel often unwinds sequential collagen crosslink as well as the combination of aggrecan and GAGs. Thus, though decellularization process would not significantly alter the elastic modulus of native AF (Sharabi et al., 2019; Xu et al., 2014; from 14.71 ± 1.19 to 34.94 ± 3.53 MPa), generating an injectable matrix is likely to reduce modulus in a large extent (Ghorbani et al., 2017). How to revert ideal mechanical properties of injectable matrix after self‐healing process is the main concern to produce such biomaterials. There are many approaches to enhance the mechanical characteristics of injectable matrix. For example, appropriate ratio of various components (Wiltsey et al., 2013), supplementation of crosslinkers (Cruz et al., 2017), and so forth are all promising approaches for enhancing mechanical features. Genipin, a broadly utilized and efficient synthetic chemical, has been proven to have the ability to improve mechanical properties of various biomaterials (Frauchiger et al., 2018). According to our findings, the modulus of DAF was gradually enhanced, as genipin concentration increased 2018. However, when genipin concentration reached 0.04%, potential cytotoxicity was observed. Therefore, 0.02% genipin could endow DAF‐G with enhanced mechanical characteristics and did not impact its biocompatibility.

The mechanism of self‐healing process of decellularized matrix is a maze full of mystery. Chemical covalent bonding (disulfide bonds, acylhydrazone bonds, DA “click chemistry”, imine bonds, etc.) as well as physical noncovalent crosslinking (hydrophobic interactions, hydrogen bonds, π‐π stacking, etc.) might be involved in the self‐healing process (Tu et al., 2019). Genipin was likely to promote the formation of secondary amide, which resulted from reaction between ester group of genipin and the amino group of chitosan (Ubaid & Murtaza, 2018). According to our findings, acylhydrazone bonds were significantly increased, whereas the alcohol group lessened, suggesting that chemical reaction between ester and amino groups occurred in this self‐healing process.

Because outstretched cellular morphology of AF cells is the primary characteristic for cells appropriate function, such as cell–matrix interaction, matrix synthesis, mechanosensors, and so forth (Bruehlmann, Rattner, Matyas, & Duncan, 2002; Errington, Puustjarvi, White, Roberts, & Urban, 1998), ideal biomaterials should allow for the formation of certain cellular morphology, when cells are planted and proliferated within those materials. Many reports have discussed the suitable portion of genipin to matrix, which significantly affects cellular extension and proliferation. By mixing various concentration of genipin to DAF matrix, we identified the 0.02% genipin that exerted undamaged biocompatibility, suitable mechanical properties, and well cells stretch.

Though many researches have investigated biomaterial‐induced AF cell‐like differentiation (Chu et al., 2019; Ma et al., 2018), no final standards have been clarified, considering the specific AF cells phenotype markers. Col I is the primary component of AF tissues, and COL1A1 is also the most broadly used AF cells gene markers (Li et al., 2019). Comparing AF cells, NP cells, and chondrocytes, gene expression profiling based on large‐scale statistics has recommended COL5A1 as a potential AF marker (Clouet et al., 2009; Lee et al., 2007). Apart from the traditional AF markers, recent transcriptional profiling based on human IVD reported that IBSP, TNMD, and FBLN1 showed higher expression level in human AF cells than NP cells (Minogue, Richardson, Zeef, Freemont, & Hoyland, 2010). Therefore, we included COL1A1, COL5A1, IBSP, FBLN1, and TNMD as potential mature AF cells markers to evaluate the capacity of biomaterials for directional differentiation. Interestingly, when cells were cultured on the hydrogels, all expression levels of all these five genes were significantly elevated in DAF‐G group, compared with Col I group, indicating enhanced AF cell‐like differentiation induced by DAF‐G, whereas genipin crosslinking did not impair or even enhance DAF‐G's ability to direct specific differentiation.

Annular defects mode has been applied as a common animal mode in many researches (Moriguchi et al., 2018). Defects in AF lead to uneven stress on the lamellae structure and continuous leakage of NP tissues on the defect area, resulting in a loss of tissue functionality and disc height. In our study, g‐DAF‐G was injected to the defect area immediately after disc injury caused by needle puncture. Injected DAF‐G and g‐DAF‐G were both well attached to the AF defect. The outcomes suggested that g‐DAF‐G group showed better results on the eighth week than the DAF‐G group. The reason might be that the mechanical properties of g‐DAF‐G were significantly higher than DAF‐G, which allowed for higher stability and better local anchor of g‐DAF‐G on defect area. More interestingly, the hydrogels filled with disc cells and abundant ECM were clearly identified on the AF defects, which indicated that the in situ regeneration was induced by DAF‐G and g‐DAF‐G.

Graft cell‐based transplantation has also been widely used in many cases of IVDD (Zhang et al., 2015). However, for lack of donors, the acquisition of autologous and allogeneic cells might be difficult. It will be hard to ensure quality of cells, and many efforts will be required for ethical review. Therefore, applying cells delivery biomaterials for clinical use still has a long way to go. What is more, although stem cell delivery with DAF‐G might show a better result in the repair of AF defect, cell‐based therapy is not a very convenient and highly operable way to induce regeneration. An alternative and attractive strategy for regenerative medicine is to target endogenous repair mechanisms in resident cell populations to induce in situ regeneration (Clarke et al., 2018). The outcomes suggested that the decellularized matrix provided hospitable cell niches and the biochemical cues for resident cells' migration and growth (D'Este, Eglin, & Alini, 2018). This endows decellularized matrix hydrogel with the natural advantages to induce in situ regeneration in IVDs. Taken all together, genipin‐crosslinked DAF‐G can serve as a promising therapeutic alternative in the repair of AF injuries or tears.

## CONFLICT OF INTEREST

The authors declare that they have no competing interests.

## AUTHORS' CONTRIBUTIONS

Yizhong Peng, Donghua Huang, and Jinye Li contributed equally to this work and shall share the first authorship. Zengwu Shao and Xiangcheng Qing contributed to the conception and design of this study. Yizhong Peng and Donghua Huang arranged the technical route. Yizhong Peng and Jinye Li performed most of the experiments, analyses, and interpretations. Sheng Liu and Jinye Li drafted the paper. Donghua Huang composed the figures and tables. Yizhong Peng and Donghua Huang substantially revised the paper. Zengwu Shao and Xiangcheng Qing gave final approval of the version to be submitted.

## Supporting information


**Figure S1.** General schematic diagram of genipin crosslinked decellularized AF hydrogels developed for intervertebral disc regeneration. AF, annulus fibrosus; DAF, decellularized annulus fibrosus; BMSCs, bone marrow mesenchymal stem cells; g‐DAF‐G, genipin crosslinked decellularized annulus fibrosus hydrogels.

**Figure S2.** Histological staining of FAF and DAF‐P. immunohistochemical staining of COL1A1 and COL2A1, picrosirius red (PSR) and masson staining were performed to evaluate the influence of decellularization on collagens. FAF, fresh annulus fibrosus. DAF‐P, decellularized annulus fibrosus powder. Scale bar = 100μm.

**Table S1.** RNA quantification of each group.
