# Supplementary figures and images for: Genipin‐crosslinked decellularized annulus fibrosus hydrogels induces tissue‐specific differentiation of bone mesenchymal stem cells and intervertebral disc regeneration
Source: J Tissue Eng Regen Med. 2020 Feb 12;14(3):497–509. doi: 10.1002/term.3014 (PMC7155128; doi:10.1002/term.3014)

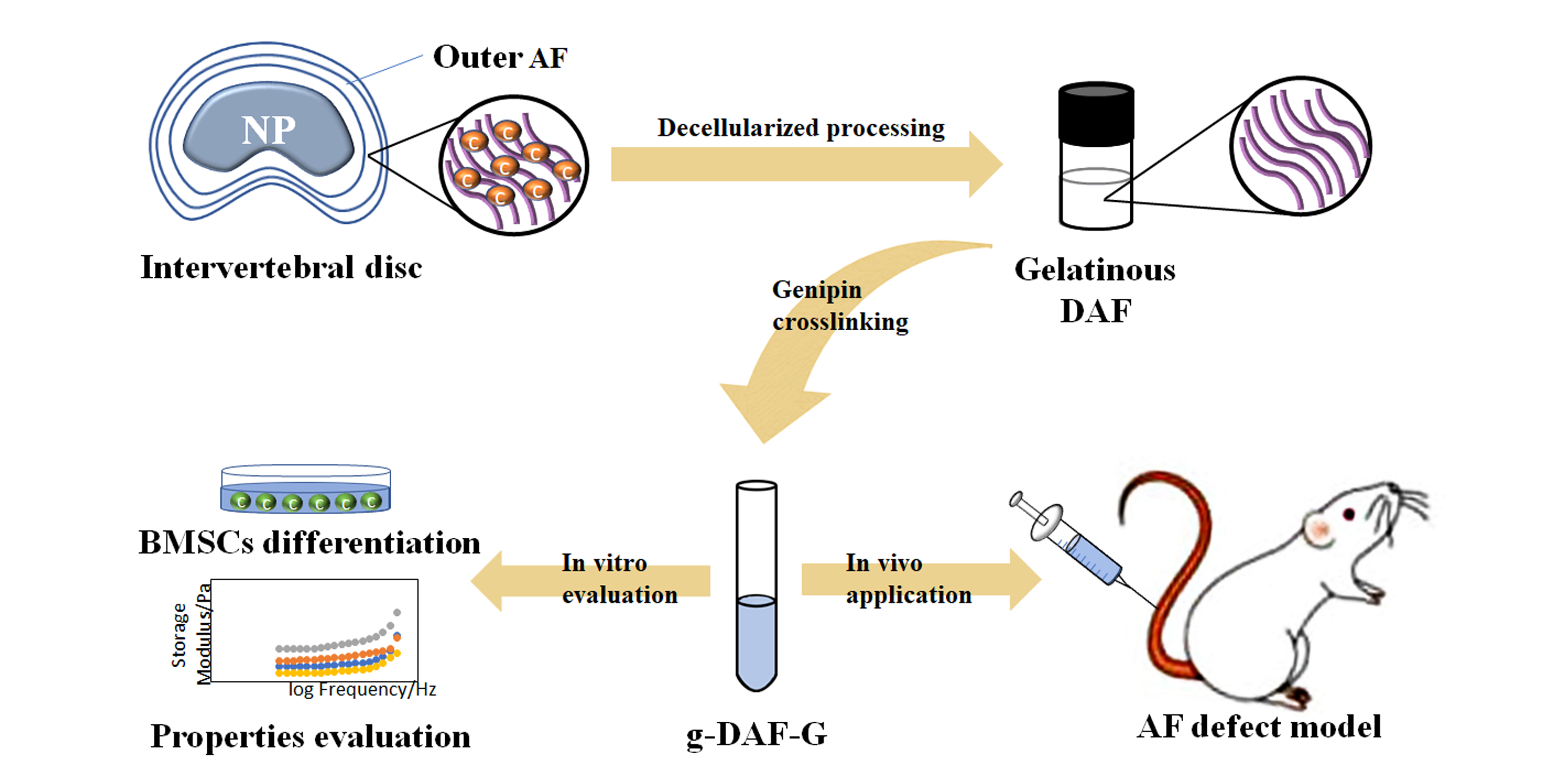

Supplement: Supplementary file 1 — Figure S1. General schematic diagram of genipin crosslinked decellularized AF hydrogels developed for intervertebral disc regeneration. AF, annulus fibrosus; DAF, decellularized annulus fibrosus; BMSCs, bone marrow mesenchymal stem cells; g‐DAF‐G, genipin crosslinked decellularized annulus fibrosus hydrogels. [file TERM-14-497-s001.tif]

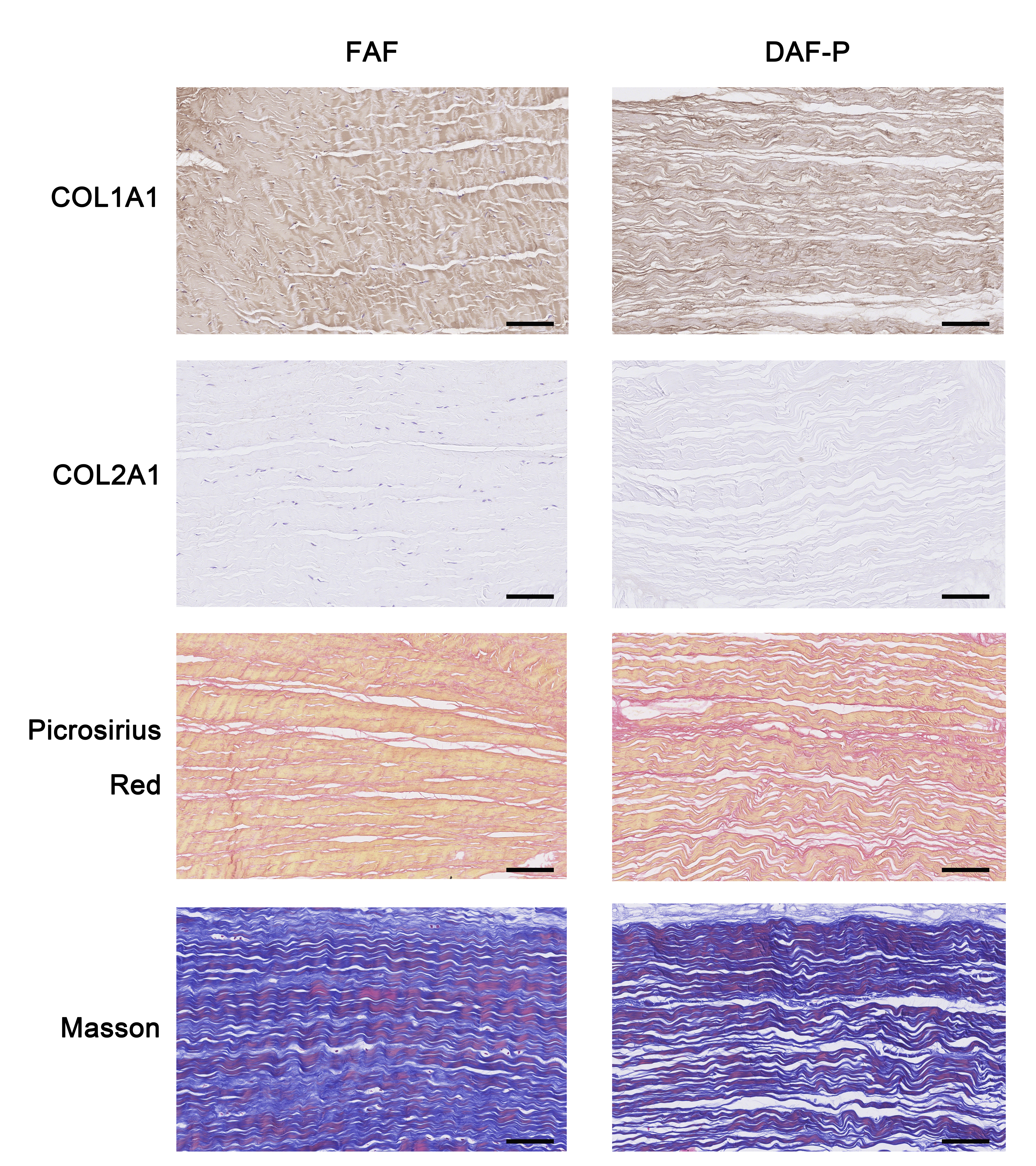

Supplement: Supplementary file 2 — Figure S2. Histological staining of FAF and DAF‐P. immunohistochemical staining of COL1A1 and COL2A1, picrosirius red (PSR) and masson staining were performed to evaluate the influence of decellularization on collagens. FAF, fresh annulus fibrosus. DAF‐P, decellularized annulus fibrosus powder. Scale bar = 100μm. [file TERM-14-497-s002.tif]
