# Supplementary material for: Genipin‐crosslinked decellularized annulus fibrosus hydrogels induces tissue‐specific differentiation of bone mesenchymal stem cells and intervertebral disc regeneration
Source: J Tissue Eng Regen Med. 2020 Feb 12;14(3):497–509. doi: 10.1002/term.3014 (PMC7155128; doi:10.1002/term.3014)
Supplement: Supplementary file 3 — Table S1. RNA quantification of each group. [file TERM-14-497-s003.docx]

| Supplementary table 1. RNA quantification of each group. | | | | | | | |
| --- | --- | --- | --- | --- | --- | --- | --- |
| **Sample ID** | **Nucleic Acid** | **Unit** | **A260 (Abs)** | **A280 (Abs)** | **260/280** | **260/230** | **Volume/ul** |
| col1-1 | 1086.9 | ng/µl | 27.172 | 13.733 | 1.98 | 2.00 | 30 |
| col1-2 | 569.8 | ng/µl | 14.246 | 7.276 | 1.96 | 1.99 | 50 |
| col1-3 | 436.5 | ng/µl | 10.912 | 5.544 | 1.97 | 1.83 | 50 |
| DAF-1 | 546.8 | ng/µl | 13.669 | 6.958 | 1.96 | 1.99 | 50 |
| DAF-2 | 478.8 | ng/µl | 11.97 | 6.307 | 1.9 | 1.93 | 50 |
| DAF-3 | 389.2 | ng/µl | 9.729 | 5.12 | 1.9 | 1.34 | 50 |
| g-DAF-1 | 564.7 | ng/µl | 14.117 | 7.278 | 1.94 | 1.85 | 50 |
| g-DAF-2 | 367.8 | ng/µl | 9.194 | 4.721 | 1.95 | 1.38 | 50 |
| g-DAF-3 | 612.1 | ng/µl | 15.303 | 7.881 | 1.94 | 1.58 | 50 |
